# A Comparison Study on Similarity and Dissimilarity Measures in Clustering Continuous Data

**DOI:** 10.1371/journal.pone.0144059

**Published:** 2015-12-11

**Authors:** Ali Seyed Shirkhorshidi, Saeed Aghabozorgi, Teh Ying Wah

**Affiliations:** 1 Department of Information Systems, Faculty of Computer Science and Information Technology, University of Malaya, 50603, Kuala Lumpur, Malaysia; 2 IBM Analytics, Platform, Emerging Technologies, IBM Canada Ltd., Markham, Ontario L6F 1C7, Canada; University of Westminster, UNITED KINGDOM

## Abstract

Similarity or distance measures are core components used by distance-based clustering algorithms to cluster similar data points into the same clusters, while dissimilar or distant data points are placed into different clusters. The performance of similarity measures is mostly addressed in two or three-dimensional spaces, beyond which, to the best of our knowledge, there is no empirical study that has revealed the behavior of similarity measures when dealing with high-dimensional datasets. To fill this gap, a technical framework is proposed in this study to analyze, compare and benchmark the influence of different similarity measures on the results of distance-based clustering algorithms. For reproducibility purposes, fifteen publicly available datasets were used for this study, and consequently, future distance measures can be evaluated and compared with the results of the measures discussed in this work. These datasets were classified as low and high-dimensional categories to study the performance of each measure against each category. This research should help the research community to identify suitable distance measures for datasets and also to facilitate a comparison and evaluation of the newly proposed similarity or distance measures with traditional ones.

## Introduction

One of the biggest challenges of this decade is with databases having a variety of data types. Variety is among the key notion in the emerging concept of big data, which is known by the 4 Vs: Volume, Velocity, Variety and Variability [1,2]. Currently, there are a variety of data types available in databases, including: interval-scaled variables (salary, height), binary variables (gender), categorical variables (religion: Jewish, Muslim, Christian, etc.) and mixed type variables (multiple attributes with various types). Despite data type, the distance measure is a main component of distance-based clustering algorithms. Partitioning algorithms, such as k-means, k-medoids and more recently soft clustering approaches for instance fuzzy c-means [3] and rough clustering [4], are mainly dependent on distance measures to recognize clusters in a dataset.

In data mining, ample techniques use distance measures to some extent. Clustering is a well-known technique for knowledge discovery in various scientific areas, such as medical image analysis [5–7], clustering gene expression data [8–10], investigating and analyzing air pollution data [11–13], power consumption analysis [14–16], and many more fields of study. Improving clustering performance has always been a target for researchers. Since in distance-based clustering similarity or dissimilarity (distance) measures are the core algorithm components, their efficiency directly influences the performance of clustering algorithms. These algorithms use similarity or distance measures to cluster similar data points into the same clusters, while dissimilar or distant data points are placed into different clusters. Examples of distance-based clustering algorithms include partitioning clustering algorithms, such as k-means as well as k-medoids and hierarchical clustering [17].

Although there are various studies available for comparing similarity/distance measures for clustering numerical data, but there are two difference between this study and other existing studies and related works: first, the aim in this study is to investigate the similarity/distance measures against low dimensional and high dimensional datasets and we wanted to analyse their behaviour in this context. Second thing that distinguish our study from others is that our datasets are coming from a variety of applications and domains while other works confined with a specific domain. In essence, the target of this research is to compare and benchmark similarity and distance measures for clustering continuous data to examine their performance while they are applied to low and high-dimensional datasets. For the sake of reproducibility, fifteen publicly available datasets [18,19] were used for this study, so future distance measures could consequently be evaluated and compared with the results of traditional measures discussed in this study. These datasets are classified into low and high-dimensional, and each measure is studied against each category. But before doing the study on similarity or dissimilarity measures, it needs to be clarified that they have significant influence on clustering quality and are worthwhile to be studied. In sections 3 (methodology) it is elaborated that the similarity or distance measures have significant influence on clustering results.

The key contributions of this paper are as follows:

- Twelve similarity measures frequently used for clustering continuous data from various fields are compiled in this study to be evaluated in a single framework. Most of these similarity measures have not been examined in domains other than the originally proposed one.
- A technical framework is proposed in this study to analyze, compare and benchmark the influence of different similarity measures on the result of distance-based clustering algorithms.
- Similarity measures are evaluated on a wide variety of publicly available datasets. Particularly, we evaluate and compare the performance of similarity measures for continuous data against datasets with low and high dimension.

The rest of paper is organized as follows: in section 2, a background on distance measures is discussed. In section 3, we have explained the methodology of the study. Experimental results with a discussion are represented in section 4, and section 5 summarizes the contributions of this study.

## Background on Distance Measures for Continuous Data

Utilization of similarity measures is not limited to clustering, but in fact plenty of data mining algorithms use similarity measures to some extent. To reveal the influence of various distance measures on data mining, researchers have done experimental studies in various fields and have compared and evaluated the results generated by different distance measures. Although it is not practical to introduce a “Best” similarity measure or a best performing measure in general, a comparison study could shed a light on the performance and behavior of measures. For instance, Boriah et al. conducted a comparison study on similarity measures for categorical data and evaluated similarity measures in the context of outlier detection for categorical data [20]. It was concluded that the performance of an outlier detection algorithm is significantly affected by the similarity measure. In their research, it was not possible to introduce a best performing similarity measure, but they analyzed and reported the situations in which a measure has poor or superior performance. In another research work, Fernando et al. [21] reviewed, compared and benchmarked binary-based similarity measures for categorical data. With some cases studies, Deshpande et al. focused on data from a single knowledge area, for example biological data, and conducted a comparison in favor of profile similarity measures for genetic interaction networks. They concluded that the Dot Product is consistent among the best measures in different conditions and genetic interaction datasets [22].

Similarly, in the context of clustering, studies have been done on the effects of similarity measures., In one study Strehl and colleagues tried to recognize the impact of similarity measures on web clustering [23]. In another, six similarity measure were assessed, this time for trajectory clustering in outdoor surveillance scenes [24]. In chemical databases, Al Khalifa et. al. [25] examined performance of twelve coefficients for clustering, similarity searching and compound selection. From the results they concluded that no single coefficient is appropriate for all methodologies.

Despite these studies, no empirical analysis and comparison is available for clustering continuous data to investigate their behavior in low and high dimensional datasets. At the other hand our datasets are coming from a variety of applications and domains and while they are limited with a specific domain. In this study, we gather known similarity/distance measures available for clustering continuous data, which will be examined using various clustering algorithms and against 15 publicly available datasets. It is not possible to introduce a perfect similarity measure for all kinds of datasets, but in this paper we will discover the reaction of similarity measures to low and high-dimensional datasets. The similarity measures with the best results in each category are also introduced.

Before presenting the similarity measures for clustering continuous data, a definition of a clustering problem should be given. Assuming that the number of clusters required to be created is an input value k, the clustering problem is defined as follows [26]:

### Definition 1

Given a dataset *D* = {*v*_1_, *v*_2_, …, *v*_*n*_} of data vectors and an integer value *k*, the clustering problem is to define a mapping *f*: *D* → {1, …, *k*} where each *v*_*i*_ is assigned to one cluster *C*_*j*_, 1 ≤ *j* ≤ *k*. A cluster *C*_*j*_ contains precisely those data vectors mapped to it; that is, *C*_*j*_ = {*v*_*i*_ | *f*(*t*_*i*_) = *C*_*j*_, 1 ≤ *i* ≤ *n*, *and v*_*i*_ ∈ *D*}.

In the rest of this study, *v*_1_, *v*_2_ represent two data vectors defined as *v*_1_ = {*x*_1_, *x*_2_, …, *x*_*n*_}, *v*_2_ = {*y*_1_, *y*_2_, …, *y*_*n*_}, where *x*_*i*_, *y*_*i*_ are called attributes.

Subsequently, similarity measures for clustering continuous data are discussed. Some of these similarity measures are frequently employed for clustering purposes while others have scarcely appeared in literature.

### Minkowski

The Minkowski family includes Euclidean distance and Manhattan distance, which are particular cases of the Minkowski distance [27–29]. The Minkowski distance is defined by $d_{min}={\left(\sum_{i=1}^{n}{\left|x_{i}-y_{i}\right|}^{m}\right)}^{\frac{1}{m}},\;m\geq 1,$ where *m* is a positive real number and *x*_*i*_ and *y*_*i*_ are two vectors in *n*-dimensional space. The Minkowski distance performs well when the dataset clusters are isolated or compacted; if the dataset does not fulfil this condition, then the large-scale attributes would dominate the others [30,31]. Another problem with Minkowski metrics is that the largest-scale feature dominates the rest. Thus, normalizing the continuous features is the solution to this problem [31].

A modified version of the Minkowski metric has been proposed to solve clustering obstacles. For example, Wilson and Martinez presented distance based on counts for nominal attributes and a modified Minkowski metric for continuous features [32].

### Manhattan distance

Manhattan distance is a special case of the Minkowski distance at m = 1. Like its parent, Manhattan is sensitive to outliers. When this distance measure is used in clustering algorithms, the shape of clusters is hyper-rectangular [33]. A study by Perlibakas demonstrated that a modified version of this distance measure is among the best distance measures for PCA-based face recognition [34]. This measure is defined as $d_{man}=\sum_{i=1}^{n}\left|x_{i}-y_{i}\right|$.

### Euclidean distance

The most well-known distance used for numerical data is probably the Euclidean distance. This is a special case of the Minkowski distance when m = 2. Euclidean distance performs well when deployed to datasets that include compact or isolated clusters [30,31]. Although Euclidean distance is very common in clustering, it has a drawback: if two data vectors have no attribute values in common, they may have a smaller distance than the other pair of data vectors containing the same attribute values [31,35,36]. Another problem with Euclidean distance as a family of the Minkowski metric is that the largest-scaled feature would dominate the others. Normalization of continuous features is a solution to this problem [31].

### Average distance

Regarding the above-mentioned drawback of Euclidean distance, average distance is a modified version of the Euclidean distance to improve the results [27,35]. For two data points x, y in *n*-dimentional space, the average distance is defined as $d_{ave}={\left(\frac{1}{n}\sum_{i=1}^{n}{\left(x_{i}-y_{i}\right)}^{2}\right)}^{\frac{1}{2}}$.

### Weighted euclidean distance

If the relative importance according to each attribute is available, then the Weighted Euclidean distance—another modification of Euclidean distance—can be used [37]. This distance is defined as $d_{we}={\left(\sum_{i=1}^{n}w_{i}{\left(x_{i}-y_{i}\right)}^{2}\right)}^{\frac{1}{2}}$, where *w*_*i*_ is the weight given to the *i*th component.

This distance measure is the only measure which is not included in this study for comparison since calculating the weights is closely related to the dataset and the aim of researcher for cluster analysis on the dataset. As an instance of using this measure reader can refer to Ji et. al. research work. They used this measure for proposing a dynamic fuzzy cluster algorithm for time series [38].

### Chord distance

Chord distance is one more Euclidean distance modification to overcome the previously mentioned Euclidean distance shortcomings. It can solve problems caused by the scale of measurements as well. Chord distance is defined as the length of the chord joining two normalized points within a hypersphere of radius one. This distance can be calculated from non-normalized data as well [27]. Chord distance is defined as $d_{chord}={\left(2-2\frac{\sum_{i=1}^{n}x_{i}y_{i}}{{\left\|x\right\|}_{2}{\left\|y\right\|}_{2}}\right)}^{\frac{1}{2}}$, where ‖*x*‖_2_ is the *L* ^2^-norm ${\left\|x\right\|}_{2}=\sqrt{\sum_{i=1}^{n}x_{i}^{2}}$.

### Mahalanobis distance

Mahalanobis distance is a data-driven measure in contrast to Euclidean and Manhattan distances that are independent of the related dataset to which two data points belong [20,33]. A regularized Mahalanobis distance can be used for extracting hyperellipsoidal clusters [30]. On the other hand, Mahalanobis distance can alleviated distortion caused by linear correlation among features by applying a whitening transformation to the data or by using the squared Mahalanobis distance [31]. Mahalanobis distance is defined by $d_{mah}=\sqrt{(x-y)S^{-1}{\left(x-y\right)}^{T}}$ where *S* is the covariance matrix of the dataset [27,39].

### Cosine deasure

The Cosine similarity measure is mostly used in document similarity [28,33] and is defined as $Cosine(x,y)=\frac{\sum_{i=1}^{n}x_{i}y_{i}}{{\left\|x\right\|}_{2}{\left\|y\right\|}_{2}}$, where ‖*y*‖_2_ is the Euclidean norm of vector *y* = (*y*_1_, *y*_2_, …, *y*_*n*_) defined as ${\left\|y\right\|}_{2}=\sqrt{y_{1}^{2}+y_{2}^{2}+\ldots +y_{n}^{2}}$. The Cosine measure is invariant to rotation but is variant to linear transformations. It is also independent of vector length [33].

### Pearson correlation

Pearson correlation is widely used in clustering gene expression data [33,36,40]. This similarity measure calculates the similarity between the shapes of two gene expression patterns. The Pearson correlation is defined by $Pearson(x,y)=\frac{\sum_{i=1}^{n}\left(x_{i}-\mu_{x})(y_{i}-\mu_{y}\right)}{\sqrt{\sum_{i=1}^{n}{\left(x_{i}-y_{i}\right)}^{2}}\sqrt{\sum_{i=1}^{n}{\left(x_{i}-y_{i}\right)}^{2}}}$, where *μ*_*x*_ and *μ*_*y*_ are the means for *x* and *y* respectively. The Pearson correlation has a disadvantage of being sensitive to outliers [33,40].

The similarity measures explained above are the most commonly used for clustering continuous data. Table 1 represents a summary of these with some highlights of each.

**Table 1:** Similarity Measures for continuous data (in time complexity, *n* is the number of dimensions of *x* and *y*).

Distance Measure	Equation	Time complexity	Advantages	Disadvantages	Applications
Euclidean Distance	$d_{euc}={\left[\sum_{i=1}^{n}{\left(x_{i}-y_{i}\right)}^{2}\right]}^{\frac{1}{2}}$	O(n)	Very common, easy to compute and works well with datasets with compact or isolated clusters [27,31].	Sensitive to outliers [27,31].	*K*-means algorithm, Fuzzy *c*-means algorithm [38].
Average Distance	$d_{ave}={\left(\frac{1}{n}\sum_{i=1}^{n}{\left(x_{i}-y_{i}\right)}^{2}\right)}^{\frac{1}{2}}$	O(n)	Better than Euclidean distance [35] at handling outliers.	Variables contribute independently to the measure of distance. Redundant values could dominate the similarity between data points [37].	*K*-means algorithm
Weighted Euclidean	$d_{we}={\left(\sum_{i=1}^{n}w_{i}{\left(x_{i}-y_{i}\right)}^{2}\right)}^{\frac{1}{2}}$	O(n)	The weight matrix allows to increase the effect of more important data points than less important one [37].	Same as Average Distance.	Fuzzy *c*-means algorithm [38]
Chord	$d_{chord}={\left(2-2\frac{\sum_{i=1}^{n}x_{i}y_{i}}{{\left\|x\right\|}_{2}{\left\|y\right\|}_{2}}\right)}^{\frac{1}{2}}$	O(3n)	Can work with un-normalized data [27].	It is not invariant to linear transformation [33].	Ecological resemblance detection [35].
Mahalanobis	$d_{mah}=\sqrt{(x-y)S^{-1}{\left(x-y\right)}^{T}}$	O(3n)	Mahalanobis is a data-driven measure that can ease the distance distortion caused by a linear combination of attributes [35].	It can be expensive in terms of computation [33]	Hyperellipsoidal clustering algorithm [30].
Cosine Measure	$Cosine(x,y)=\frac{\sum_{i=1}^{n}x_{i}y_{i}}{{\left\|x\right\|}_{2}{\left\|y\right\|}_{2}}$	O(3n)	Independent of vector length and invariant to rotation [33].	It is not invariant to linear transformation [33].	Mostly used in document similarity applications [28,33].
Manhattan	$d_{man}=\sum_{i=1}^{n}\left(x_{i}-y_{i}\right)$	O(n)	Is common and like other Minkowski-driven distances it works well with datasets with compact or isolated clusters [27].	Sensitive to the outliers.[27,31]	*K*-means algorithm
Mean Character Difference	$d_{MCD}=\frac{1}{n}\sum_{i=1}^{n}\left|x_{i}-y_{i}\right|$	O(n)	*Results in accurate outcomes using the K-medoids algorithm.	*Low accuracy for high-dimensional datasets using K-means.	Partitioning and hierarchical clustering algorithms.
Index of Association	$d_{IOA}=\frac{1}{n}\sum_{i=1}^{n}\left|\frac{x_{i}}{\sum_{i=1}^{n}x_{i}}-\frac{y_{i}}{\sum_{i=1}^{n}y_{i}}\right|$	O(3n)	-	*Low accuracy using K-means and K-medoids algorithms.	Partitioning and hierarchical clustering algorithms.
Canberra Metric	$d_{canb}=\sum_{i=1}^{n}\frac{\left|x_{i}-y_{i}\right|}{\left(x_{i}+y_{i}\right)}$	O(n)	*Results in accurate outcomes for high-dimensional datasets using the K-medoids algorithm.	-	Partitioning and hierarchical clustering algorithms.
Czekanowski Coefficient	$d_{czekan}=1-\frac{2\;\sum_{i=1}^{n}min(x_{i},y_{i})}{\sum_{i=1}^{n}\left(x_{i}+y_{i}\right)}$	O(2n)	*Results in accurate outcomes for medium-dimensional datasets using the K-means algorithm.	-	Partitioning and hierarchical clustering algorithms.
Coefficient of Divergence	$d_{canb}={\left(\frac{1}{n}\sum_{i=1}^{n}{\left(\frac{x_{i}-y_{i}}{x_{i}+y_{i}}\right)}^{2}\right)}^{\frac{1}{2}}$	O(n)	*Results in accurate outcomes using the K-means algorithm.	-	Partitioning and hierarchical clustering algorithms.
Pearson coefficient	$Pearson(x,y)=\frac{\sum_{i=1}^{n}\left(x_{i}-\mu_{x})(y_{i}-\mu_{y}\right)}{\sqrt{\sum_{i=1}^{n}{\left(x_{i}-y_{i}\right)}^{2}}\sqrt{\sum_{i=1}^{n}{\left(x_{i}-y_{i}\right)}^{2}}}$	O(2n)	*Results in accurate outcomes using the hierarchical single-link algorithm for high dimensional datasets.	-	Partitioning and hierarchical clustering algorithms.

*Points marked by asterisk are compiled based on this article’s experimental results.

## Methodology of the Study

### 3.1 Experimental design

**Fig 1:**
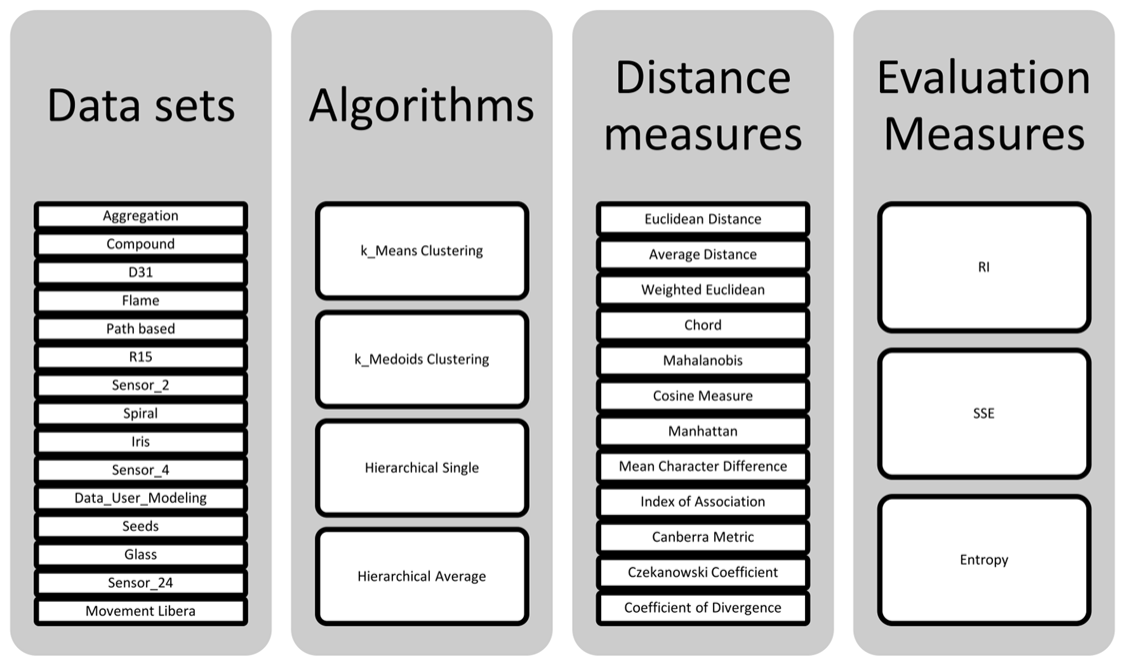
Overview of experimental study.

This section is devoted to explain the method and the framework which is used in this study for evaluating the effect of similarity measures on clustering quality. The main objective of this research study is to analyse the effect of different distance measures on quality of clustering algorithm results. As it is illustrated in Fig 1 there are 15 datasets used with 4 distance based algorithms on a total of 12 distance measures. All the distance measures in Table 1 are examined except the Weighted Euclidean distance which is dependent on the dataset and the aim of clustering.

**Fig 2:**
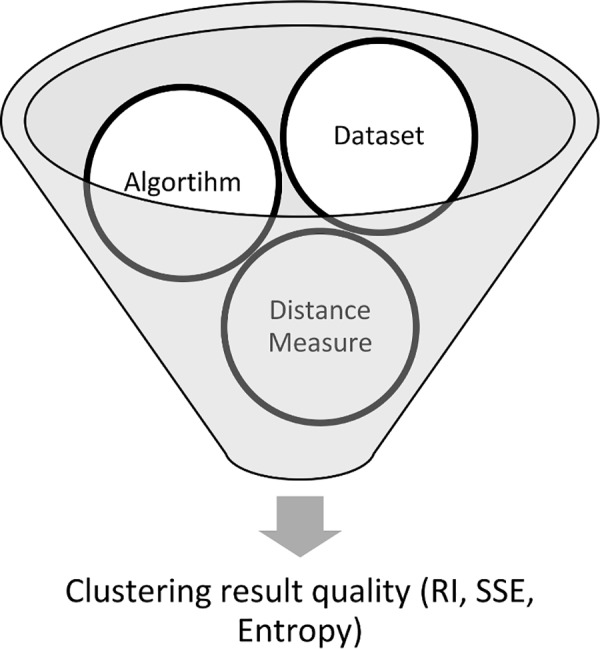
Arrangement of experiments.

Fig 2 explains the methodology of the study briefly. For each dataset we examined all four distance based algorithms, and each algorithms’ quality of clustering has been evaluated by each 12 distance measures as it is demonstrated in Fig 1. It makes a total of 720 experiments in this research work to analyse the effect of distance measures. Representing and comparing this huge number of experiments is a challenging task and could not be done using ordinary charts and tables. Consequently we have developed a special illustration method using heat mapped tables in order to demonstrate all the results in the way that could be read and understand quickly. This method is described in section 4.1.1.

### 3.2 Rand Index

In this study, we used Rand Index (RI) for evaluation of clustering outcomes resulted by various distance measures. This section is an overview on this measure and it investigates the reason that this measure has been chosen.

Rand index is frequently used in measuring clustering quality. It is a measure of agreement between two sets of objects: first is the set produced by clustering process and the other defined by external criteria. Although there are different clustering measures such as Sum of Squared Error, Entropy, Purity, Jaccard etc. but among them the Rand index is probably the most used index for cluster validation [17,41,42]. Assuming *S* = {*o*_1_, *o*_2_, …, *o*_*n*_} is a set of *n* elements and two partitions of *S* are given to compare *C* = {*c*_1_, *c*_2_, …, *c*_*r*_}, which is a partition of S into r subsets and *G* = {*g*_1_, *g*_2_, …, *g*_*s*_}, a partition of S into s subsets, the Rand index (R) is defined as follows:

### Definition 2

$$RI=\frac{a+b}{a+b+c+d}$$ 1

where:

- *a* is the number of pairs of vectors in S that are in the same set in *C* and in the same set in G.
- *b* is the number of pairs of elements in S that are in different sets in *C* and in different sets in G.
- *c* is the number of pairs of elements in S that are in the same set in *C* and in different sets in G.
- *d* is the number of pairs of elements in S that are in different sets in *C* and in the same set in G.

There is a modified version of rand index called Adjusted Rand Index (ARI) which is proposed by Hubert and Arabie [42] as an improvement for known problems with RI. These problems happen when the expected value of the RI of two random partition does not take a constant value (zero for example) or the Rand statistic approaches its upper limit of unity as the number of cluster increases. However, since our datasets don’t have these problems and also owing to the fact that the results generated using ARI were following the same pattern of RI results, we have used Rand Index in this study due to its popularity in clustering community for clustering validation.

In this study we normalized the Rand Index values for the experiments. The normalized values are between 0 and 1 and we used following formula to approach it:

$$z_{i}=\frac{r_{i}-\min\left(r\right)}{\max\left(r\right)-\min\left(r\right)}$$ 2

where *r* = (*r*_1_, …, *r*_*n*_) is the array of rand indexes produced by each similarity measure.

### 3.3 Analysis of variance (ANOVA) test

Before continuing this study, the main hypothesis needs to be proved: “distance measure has a considerable influence on clustering results”. In order to show that distance measures cause significant difference on clustering quality, we have used ANOVA test. For this purpose we will consider a null hypothesis: “distance measures doesn’t have significant influence on clustering quality”. Using ANOVA test, if the p value be very small, it means that there is very small opportunity that null hypothesis is correct, and consequently we can reject it.

ANOVA analyzes the differences among a group of variable which is developed by Ronald Fisher [43]. ANOVA is a statistical test that demonstrate whether the mean of several groups are equal or not and it can be said that it generalizes the t-test for more than two groups. It is useful for testing means of more than two groups or variable for statistical significance. Statistical significance in statistics is achieved when a p-value is less than the significance level [44]. The p-value is the probability of obtaining results which acknowledge that the null hypothesis is true [45].

For ANOVA test we have considered a table with the structure shown in Table 2 which covers all RI results for all four algorithms and each distance/similarity measure and for all datasets. Table is divided into 4 section for four respective algorithms. In each sections rows represent results generated with distance measures for a dataset.

**Table 2:** Rand Index values used for ANOVA test (in the table HAverage stands for Hierarchical Average algorithm and HSingle stands for Hierarchical Single link).

Dataset	Distance/Similarity Measures											
	Euclidean	Average	Cosine	Chord	Mahalanobis	Canberra	CoeffDiv	Czekan	IndOfAssoc	Manhattan	MCharDiff	Pearson
k-Means												
sensor_2	0.722	0.733	0.659	0.659	0.725	0.744	0.741	0.765	0.662	0.729	0.729	0.403
Aggregation	0.929	0.929	0.798	0.799	0.927	0.921	0.904	0.949	0.799	0.927	0.927	0.636
Compound	0.919	0.914	0.746	0.746	0.926	0.890	0.908	0.886	0.744	0.906	0.904	0.497
Flame	0.756	0.756	0.569	0.569	0.750	0.716	0.498	0.710	0.557	0.750	0.750	0.536
Pathbased	0.750	0.750	0.639	0.639	0.758	0.735	0.733	0.746	0.637	0.748	0.748	0.635
R15	0.999	0.999	0.949	0.948	0.999	0.999	0.998	0.998	0.947	0.998	0.998	0.552
Spiral	0.554	0.554	0.562	0.562	0.555	0.550	0.552	0.553	0.562	0.556	0.556	0.496
D31	0.994	0.992	0.956	0.956	0.995	0.992	0.992	0.994	0.956	0.994	0.994	0.528
Iris	0.880	0.880	0.966	0.966	0.880	0.942	0.950	0.927	0.958	0.874	0.874	0.776
sensor_4	0.612	0.624	0.637	0.637	0.619	0.745	0.709	0.737	0.649	0.726	0.728	0.670
Data_User_Modeling	0.725	0.725	0.668	0.668	0.719	0.711	0.706	0.713	0.668	0.712	0.711	0.657
Seeds	0.876	0.874	0.884	0.884	0.876	0.859	0.782	0.891	0.890	0.872	0.872	0.359
Glass	0.741	0.742	0.737	0.740	0.732	0.604	0.602	0.734	0.732	0.734	0.731	0.342
sensor_24	0.610	0.615	0.614	0.617	0.596	0.618	0.621	0.613	0.610	0.604	0.611	0.626
Libras movement	0.914	0.917	0.913	0.917	0.915	0.911	0.914	0.910	0.913	0.914	0.912	0.918
k-Medoids												
sensor_2	0.777	0.736	0.661	0.661	0.729	0.804	0.806	0.797	0.675	0.785	0.796	0.403
Aggregation	0.949	0.949	0.790	0.790	0.950	0.928	0.901	0.958	0.787	0.941	0.953	0.636
Compound	0.925	0.911	0.734	0.733	0.920	0.890	0.890	0.900	0.740	0.916	0.913	0.497
Flame	0.762	0.762	0.538	0.538	0.756	0.705	0.498	0.716	0.565	0.744	0.744	0.536
Pathbased	0.746	0.746	0.606	0.606	0.756	0.743	0.745	0.745	0.667	0.741	0.741	0.635
R15	0.999	0.999	0.947	0.945	0.988	0.998	0.988	0.998	0.947	0.999	0.998	0.552
Spiral	0.555	0.554	0.555	0.555	0.555	0.571	0.555	0.557	0.551	0.556	0.564	0.496
D31	0.994	0.992	0.956	0.956	0.992	0.990	0.988	0.991	0.956	0.991	0.994	0.528
Iris	0.912	0.912	0.966	0.966	0.824	0.927	0.950	0.906	0.950	0.880	0.880	0.776
sensor_4	0.707	0.711	0.711	0.711	0.656	0.740	0.722	0.709	0.690	0.696	0.716	0.656
Data_User_Modeling	0.725	0.712	0.654	0.654	0.728	0.285	0.285	0.285	0.646	0.734	0.745	0.659
Seeds	0.874	0.874	0.842	0.842	0.798	0.872	0.771	0.876	0.865	0.867	0.867	0.359
Glass	0.735	0.736	0.738	0.732	0.711	0.633	0.582	0.737	0.735	0.737	0.739	0.342
sensor_24	0.624	0.623	0.623	0.622	0.588	0.652	0.634	0.630	0.629	0.620	0.617	0.613
Libras movement	0.907	0.909	0.908	0.905	0.720	0.897	0.905	0.901	0.906	0.904	0.904	0.907
HSingle												
sensor_2	0.432	0.432	0.355	0.355	0.432	0.432	0.432	0.431	0.365	0.432	0.432	0.405
Aggregation	0.926	0.926	0.574	0.574	0.926	0.619	0.927	0.927	0.550	0.926	0.926	0.635
Compound	0.890	0.890	0.415	0.415	0.896	0.895	0.898	0.891	0.415	0.712	0.712	0.497
Flame	0.541	0.541	0.522	0.522	0.541	0.531	0.531	0.541	0.522	0.541	0.541	0.538
Pathbased	0.338	0.338	0.362	0.362	0.340	0.339	0.338	0.338	0.362	0.338	0.338	0.635
R15	0.910	0.910	0.817	0.817	0.910	0.856	0.857	0.856	0.817	0.911	0.911	0.574
Spiral	1.000	1.000	0.383	0.383	1.000	0.781	0.781	0.781	0.383	1.000	1.000	0.497
D31	0.779	0.779	0.818	0.818	0.754	0.740	0.731	0.730	0.518	0.755	0.755	0.536
Iris	0.777	0.777	0.772	0.772	0.343	0.753	0.753	0.772	0.772	0.776	0.776	0.772
sensor_4	0.341	0.341	0.345	0.345	0.346	0.451	0.339	0.333	0.345	0.338	0.338	0.651
Data_User_Modeling	0.309	0.309	0.301	0.301	0.304	0.302	0.302	0.305	0.302	0.299	0.299	0.311
Seeds	0.357	0.357	0.340	0.340	0.337	0.340	0.337	0.340	0.340	0.340	0.340	0.358
Glass	0.304	0.304	0.308	0.308	0.309	0.293	0.294	0.308	0.308	0.308	0.308	0.342
sensor_24	0.347	0.347	0.346	0.346	0.353	0.346	0.347	0.346	0.346	0.345	0.345	0.349
Libras movement	0.187	0.187	0.202	0.202	0.131	0.183	0.183	0.187	0.192	0.187	0.187	0.296
HAverage												
sensor_2	0.466	0.466	0.634	0.634	0.506	0.466	0.729	0.716	0.634	0.466	0.466	0.404
Aggregation	1.000	1.000	0.778	0.778	0.997	0.930	0.948	0.927	0.778	0.991	0.991	0.643
Compound	0.921	0.921	0.676	0.676	0.921	0.850	0.852	0.829	0.697	0.933	0.933	0.511
Flame	0.721	0.721	0.503	0.503	0.847	0.512	0.529	0.501	0.503	0.689	0.689	0.538
Pathbased	0.738	0.738	0.699	0.699	0.754	0.438	0.377	0.708	0.629	0.724	0.724	0.635
R15	0.999	0.999	0.917	0.917	0.999	0.981	0.963	0.990	0.914	0.998	0.998	0.566
Spiral	0.537	0.537	0.528	0.528	0.557	0.424	0.499	0.498	0.428	0.540	0.540	0.497
D31	0.994	0.994	0.950	0.950	0.996	0.977	0.979	0.986	0.952	0.996	0.996	0.537
Iris	0.892	0.892	0.772	0.772	0.343	0.753	0.753	0.778	0.772	0.886	0.886	0.776
sensor_4	0.338	0.338	0.561	0.561	0.338	0.479	0.479	0.480	0.544	0.376	0.376	0.653
Data_User_Modeling	0.659	0.659	0.301	0.301	0.337	0.302	0.302	0.307	0.309	0.645	0.645	0.594
Seeds	0.887	0.887	0.691	0.691	0.337	0.879	0.581	0.802	0.688	0.802	0.802	0.362
Glass	0.329	0.329	0.570	0.570	0.309	0.328	0.323	0.415	0.415	0.415	0.415	0.369
sensor_24	0.353	0.353	0.538	0.538	0.347	0.498	0.516	0.518	0.521	0.428	0.428	0.446
Libras movement	0.886	0.886	0.892	0.892	0.131	0.582	0.613	0.827	0.844	0.861	0.861	0.886

ANOVA test is performed for each algorithm separately to find if distance measures have significant impact on clustering results in each clustering algorithm.

The ANOVA test result on above table is demonstrated in the Tables 3–6.

**Table 3:** ANOVA results for k-means.

K_means	SS	df	MS	F	Prob>F
Columns	0.68317	11	0.06211	2.96	0.0013
Error	3.52624	168	0.02099		
Total	4.20942	179			

**Table 4:** ANOVA results for k-medoids.

K_medoids	SS	df	MS	F	Prob>F
Columns	0.69565	11	0.06324	2.62	0.0042
Error	4.05766	168	0.02415		
Total	4.75331	179			

**Table 5:** ANOVA results for HSingle.

HAvrage	SS	df	MS	F	Prob>F
Columns	0.47251	11	0.04296	2.62	0.0043
Error	2.52617	154	0.0164		
Total	8.91175	175			

**Table 6:** ANOVA results for HSingle.

HSingle	SS	df	MS	F	Prob>F
Columns	0.3194	11	0.02903	2.38	0.0095
Error	1.8788	154	0.0122		
Total	10.2233	179			

The small Prob values indicates that differences between means of the columns are significant. From that we can conclude that the similarity measures have significant impact in clustering quality. In the rest of this study we will inspect how these similarity measures influence on clustering quality.

## Experimental Results

It is noted that references to all data employed in this work are available in acknowledgment section. A diverse set of similarity measures for continuous data was studied on low and high-dimensional continuous datasets in order to clarify and compare the accuracy of each similarity measure in different datasets with various dimensionality situations and using 15 datasets [18,19,46–49]. Details of the datasets applied in this study are represented in Table 7.

**Table 7:** Dataset Details.

Dataset Name	Dimensions	Clusters	Vectors
Aggregation	2	7	788
Compound	2	6	399
D31	2	31	3100
Flame	2	2	240
Path based	2	3	300
R15	2	15	600
Sensor_2	2	4	5456
Spiral	2	3	312
Iris	4	3	150
Sensor_4	4	4	5456
Data_User_Modeling	5	4	258
Seeds	7	3	210
Glass	9	7	214
Sensor_24	24	4	5456
Movement Libera	90	15	360

The experiments were conducted using partitioning (k-means and k-medoids) and hierarchical algorithms, which are distance-based. As it is discussed in section 3.2 the Rand index served to evaluate and compare the results. The results for each of these algorithms are discussed later in this section.

The k-means and k-medoids algorithms were used in this experiment as partitioning algorithms, and the Rand index served accuracy evaluation purposes. Due to the fact that the k-means and k-medoids algorithm results are dependent on the initial, randomly selected centers, and in some cases their accuracy might be affected by local minimum trap, the experiment was repeated 100 times for each similarity measure, after which the maximum Rand index was considered for comparison.

### 4.1 Illustration technique

**Fig 3:**
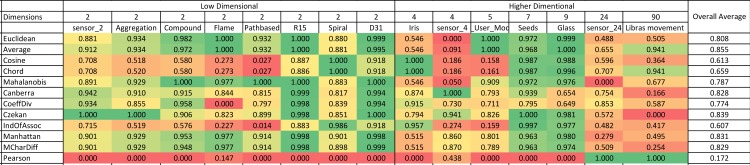
K-means color scale table for normalized Rand index values (green represents the highest and it changes to red, which is the lowest Rand index value).

**Fig 4:**
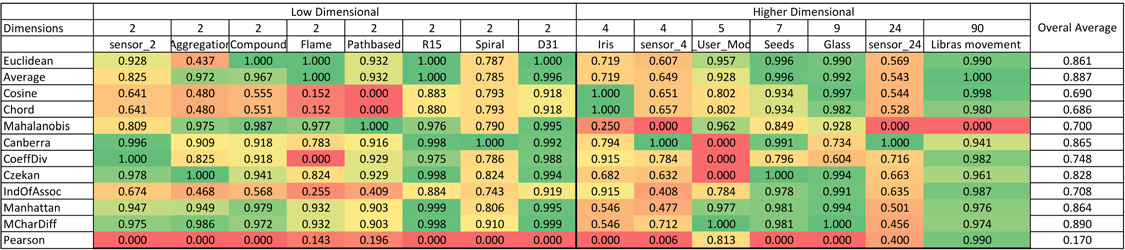
K-medoids color scale table for normalized Rand index values (green is the highest and changes color to red, which is the lowest Rand index value).

A summary of the normalized Rand index results is illustrated in color scale tables in Fig 3 and Fig 4. Since the aim of this study is to investigate and evaluate the accuracy of similarity measures for different dimensional datasets, the tables are organized based on horizontally ascending dataset dimensions. After the first column, which contains the names of the similarity measures, the remaining table is divided in two batches of columns (low and high-dimensional) that demonstrate the normalized Rand indexes for low and high-dimensional datasets, respectively. The final column considered in this table is ‘overall average’ in order to explore the most accurate similarity measure in general. This illustrational structure and approach is used for all four algorithms in this paper.

### 4.2 Benchmarking similarity measures for partitioning algorithms

Fig 3 represents the results for the k-means algorithm. According to the figure, for low-dimensional datasets, the Mahalanobis measure has the highest results among all similarity measures. On the other hand, for high-dimensional datasets, the Coefficient of Divergence is the most accurate with the highest Rand index values. Fig 4 provides the results for the k-medoids algorithm. Mean Character Difference is the most precise measure for low-dimensional datasets, while the Cosine measure represents better results in terms of accuracy for high-dimensional datasets. Overall, Mean Character Difference has high accuracy for most datasets.

As a general result for the partitioning algorithms used in this study, average distance results in more accurate and reliable outcomes for both algorithms. It is the most accurate measure in the k-means algorithm and at the same time, with very little difference, it stands in second place after Mean Character Difference for the k-medoids algorithm.

**Fig 5:**
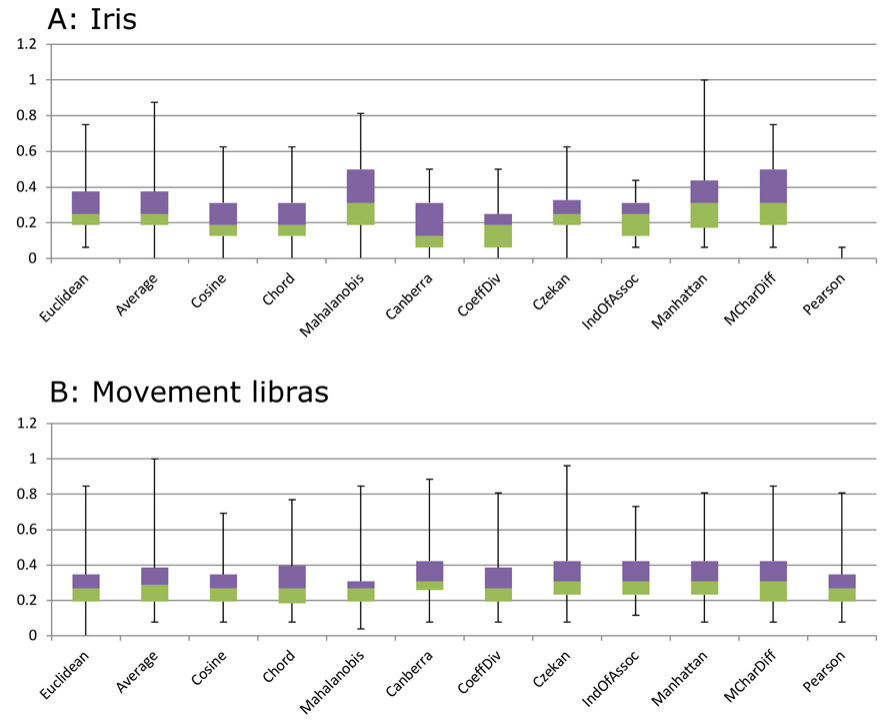
Sample box charts for k-means iteration counts created with a collection of normalized results after 100 times of repeating the algorithm for each similarity measure and dataset.

From another perspective, similarity measures in the k-means algorithm can be investigated to clarify which would lead to the k-means converging faster. However the convergence of k-means and k-medoid algorithms is not guaranteed due to the possibility of falling in local minimum trap. For this reason we have run the algorithm 100 times to prevent bias toward this weakness. Fig 5 shows two sample box charts created by using normalized data, which represents the normalized iteration count needed for the convergence of each similarity measure. Results were collected after 100 times of repeating the k-means algorithm for each similarity measure and dataset.

**Fig 6:**
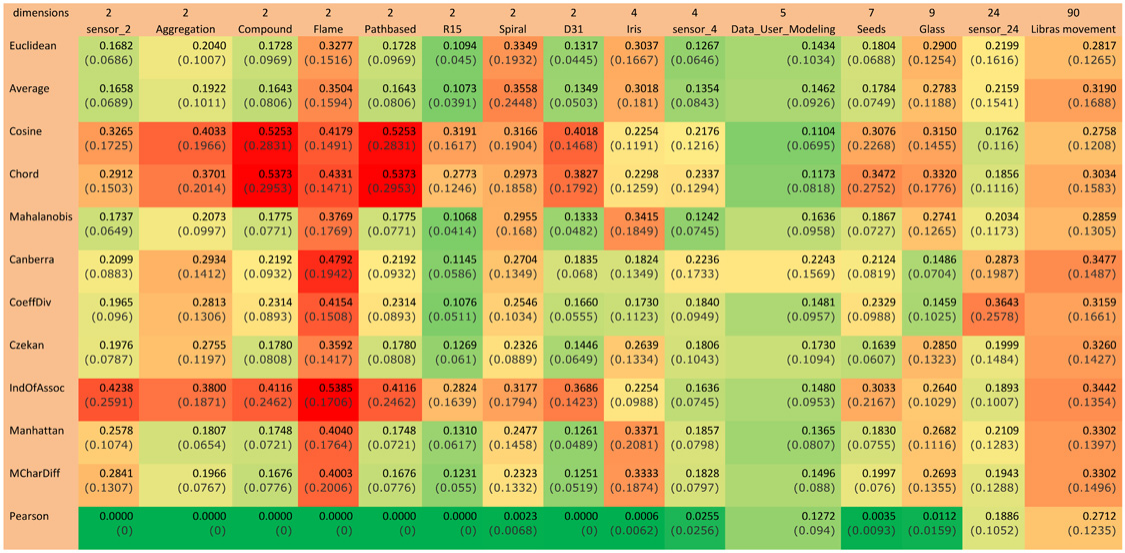
Color scale table for iteration count mean and variance (green is the lowest and it changes color to red, which shows the greatest iteration count value).

Fig 6 is a summarized color scale table representing the mean and variance of iteration counts for all 100 algorithm runs. Pearson has the fastest convergence in most datasets. After Pearson, Average is the fastest similarity measure in terms of convergence.

Regarding the discussion on Rand index and iteration count, it is manifested that the Average measure is not only accurate in most datasets and with both k-means and k-medoids algorithms, but it is the second fastest similarity measure after Pearson in terms of convergence, making it a secure choice when clustering is necessary using k-means or k-medoids algorithms.

### 4.3 Benchmarking similarity measures for hierarchical algorithms

**Fig 7:**
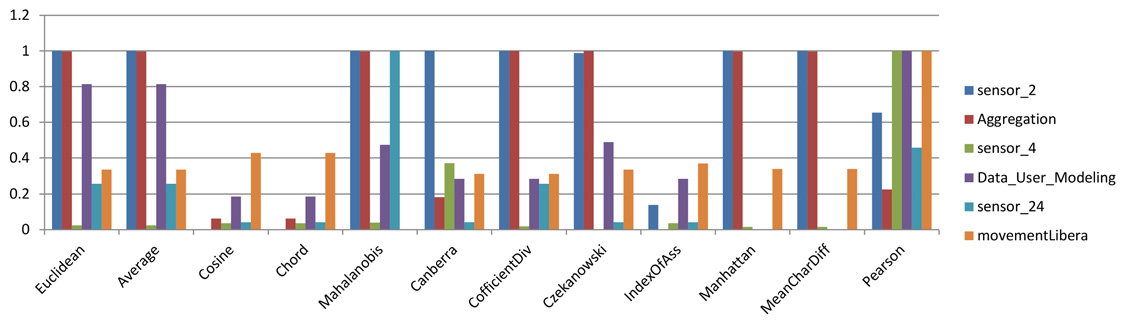
Bar chart of normalized Rand index values for selected datasets using the Single-link algorithm.

**Fig 8:**
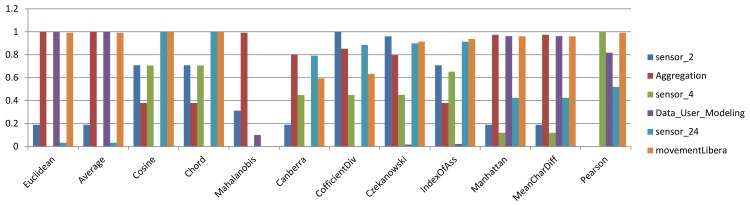
Bar chart of normalized Rand index values for selected datasets using the Group Average algorithm.

**Fig 9:**
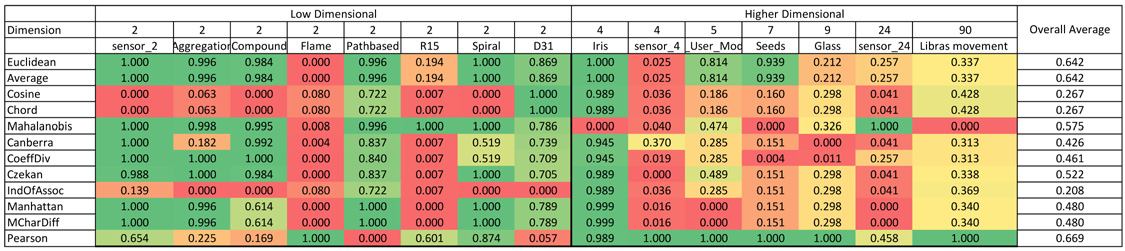
Color scale table of normalized Rand index values for the Single-link method (green is the highest and it changes color to red, which represents the lowest Rand index value).

**Fig 10:**
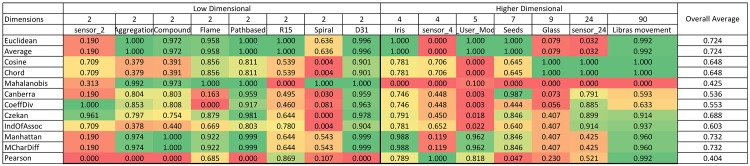
Color scale table of normalized Rand index values for Group Average (green is the highest and it changes color to red, which signifies the lowest Rand index value).

In a previous section, the influence of different similarity measures on k-means and k-medoids algorithms as partitioning algorithms was evaluated and compared. In this section, the results for Single-link and Group Average algorithms, which are two hierarchical clustering algorithms, will be discussed for each similarity measure in terms of the Rand index. Fig 7 and Fig 8 represent sample bar charts of the results. The bar charts include 6 sample datasets. Because bar charts for all datasets and similarity measures would be jumbled, the results are presented using color scale tables for easier understanding and discussion. As discussed in the last section, Fig 9 and Fig 10 are two color scale tables that demonstrate the normalized Rand index values for each similarity measure. The results in Fig 9 for Single-link show that for low-dimensional datasets, the Mahalanobis distance is the most accurate similarity measure and Pearson is the best among other measures for high-dimensional datasets. The overall average column in this figure shows that generally, Pearson presents the highest accuracy and the Average and Euclidean distances are among the most accurate measures. For the Group Average algorithm, as seen in Fig 10, Euclidean and Average are the best among all similarity measures for low-dimensional datasets. For high-dimensional datasets, Cosine and Chord are the most accurate measures. Generally, in the Group Average algorithm, Manhattan and Mean Character Difference have the best overall Rand index results followed by Euclidean and Average. Considering the overall results, it is clear that the Average measure is constantly among the best measures, and for both Single-link and Group Average algorithms.

A review of the results and discussions on the k-means, k-medoids, Single-link and Group Average algorithms reveals that by considering the overall results, the Average measure is regularly among the most accurate measures for all four algorithms.

According to heat map tables it is noticeable that Pearson correlation is behaving differently in comparison to other distance measures. It specially shows very weak results with centroid based algorithms, k-means and k-medoids. Based on the results in this research, in general, Pearson correlation doesn’t work properly for low dimensional datasets while it shows better results for high dimensional datasets.

**Fig 11:**
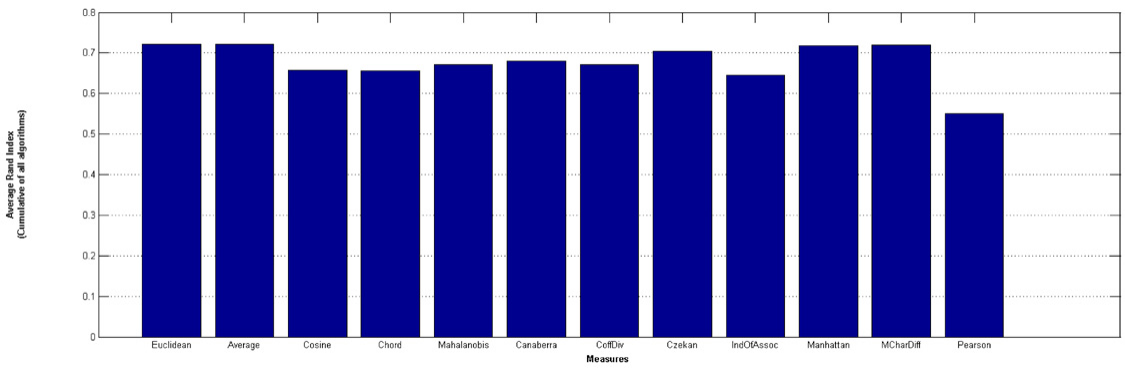
Overall RI Average.

**Fig 12:**
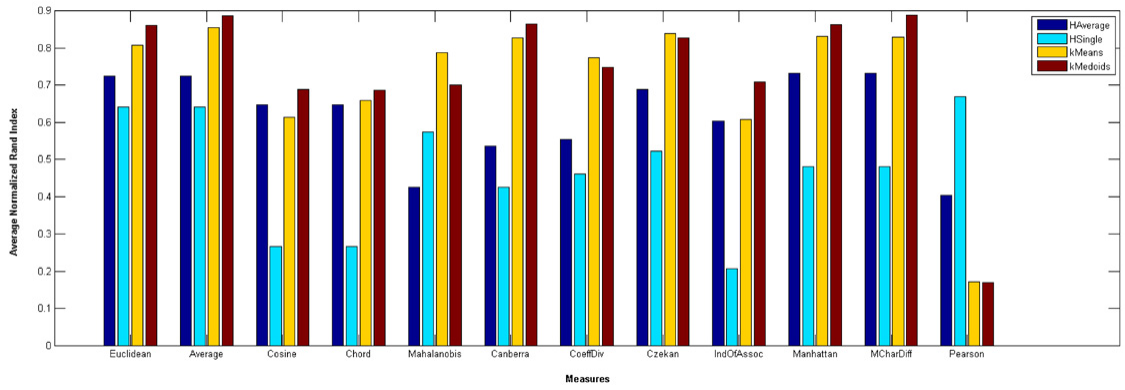
Average RI for four algorithms.

Fig 11 illustrates the overall average RI in all 4 algorithms and all 15 datasets also uphold the same conclusion. Fig 12 at the other hand shows the average RI for 4 algorithms separately. It can be inferred that Average measure among other measures is more accurate.

Furthermore, by using the k-means algorithm, this similarity measure is the fastest after Pearson in terms of convergence.

## Concluding Remarks

Selecting the right distance measure is one of the challenges encountered by professionals and researchers when attempting to deploy a distance-based clustering algorithm to a dataset. The variety of similarity measures can cause confusion and difficulties in choosing a suitable measure. Similarity measures may perform differently for datasets with diverse dimensionalities. The aim of this study was to clarify which similarity measures are more appropriate for low-dimensional and which perform better for high-dimensional datasets in the experiments. In this work, similarity measures for clustering numerical data in distance-based algorithms were compared and benchmarked using 15 datasets categorized as low and high-dimensional datasets. The accuracy of similarity measures in terms of the Rand index was studied and the best similarity measures for each of the low and high-dimensional datasets were discussed for four well-known distance-based algorithms. Overall, the results indicate that Average Distance is among the top most accurate measures for all clustering algorithms employed in this article. Moreover, this measure is one of the fastest in terms of convergence when k-means is the target clustering algorithm. Based on results in this study, in general, Pearson correlation is not recommended for low dimensional datasets. It also is not compatible with centroid based algorithms. However, this measure is mostly recommended for high dimensional datasets and by using hierarchical approaches.
